# Differential Associations of Healthy Lifestyle Latent Classes with Glycemic Transitions in Prediabetes: An Observational Study Based on CHARLS

**DOI:** 10.3390/healthcare14182966

**Published:** 2026-09-11

**Authors:** Minqi Ma, Xueqin Ma, Huoling Pan, Weibo Lyu, Fan Zhang, Jinyu Zhang

**Affiliations:** 1 School of Nursing, Shanghai University of Traditional Chinese Medicine, Shanghai 201203, China; 15801805237@163.com (M.M.); phl202405@163.com (H.P.); lyu_weibo@shutcm.edu.cn (W.L.); 2 Department of Endocrinology II, First Affiliated Hospital of Kunming Medical University, Kunming 650032, China; yxthmxq@126.com; 3 Department of Nephrology, Longhua Hospital, Shanghai University of Traditional Chinese Medicine, Shanghai 200032, China; fanzhang@shutcm.edu.cn

**Keywords:** prediabetes, lifestyle, disease progression, risk factors

## Abstract

**Highlights:**

**What are the main findings?**

- Over a four-year follow-up, among older adults with prediabetes in China, the patterns of glycemic transition were as follows: 1644 individuals (65%) remained in the prediabetic range, 322 (13%) progressed to diabetes, and 563 (22%) returned to normal glucose levels.
- Three distinct latent classes of healthy lifestyles were identified among older adults with prediabetes—smoking and drinking (27.6%), high-BMI (28.2%), and healthy lifestyle group (44.3%). These latent classes provide a reference for classifying health-risk profiles.
- Compared with the healthy lifestyle group, the odds ratios for reverting to normoglycemia were 0.736 and 0.628 for the smoking and drinking group and the high-BMI group, respectively, and the odds ratio (OR) for progression to diabetes in the high-BMI group was 2.17.

**What are the implications of the main findings?**

- In the prediabetes population, healthy behaviour clustering patterns were identified, and three latent classes were distinguished. This finding suggests that health behaviours among individuals with prediabetes do not exist in isolation; they exhibit intrinsic clustering, and traditional single-dimension classifications or scoring methods may mask the population’s true phenotypic heterogeneity.
- The clustering approach used in this study provides directions for future research. Its stability and reproducibility should be validated in prospective cohorts; the drivers underlying the latent subtypes and their associations with long-term glycemic outcomes should be explored, thereby offering a methodological basis for stratified management research.

**Abstract:**

**Introduction:** Current clinical guidelines recommend comprehensive management of multiple healthy lifestyles as fundamental to prediabetes care; however, the evidence base regarding the status and clustering of these risk factors remains insufficient. We identified distinct latent classes of healthy lifestyle in individuals with prediabetes and examined their impact on glycemic progression. **Methods:** This study included 2529 baseline prediabetic participants from the China Health and Retirement Longitudinal Study (CHARLS). We performed latent class analysis (LCA) to stratify participants according to healthy lifestyle factors: physical activity, smoking, alcohol consumption, sleep duration, body mass index (BMI), and blood pressure. Multiple imputation was used for missing covariate data. Multinomial logistic regression was used to examine associations with glycemic transition. **Results:** Three healthy lifestyle classes were identified: Class 1 (27.6%, the smoking and drinking group), Class 2 (28.2%, the high-BMI group), and Class 3 (44.3%, the healthy lifestyle group). Compared with Class 3, Class 1 (OR, 0.736; 95% CI, 0.554 to 0.978) and Class 2 (OR, 0.628; 95% CI, 0.489 to 0.806) had significantly decreased odds of returning to normoglycemia. Conversely, Class 2 (OR, 2.167; 95% CI, 1.626 to 2.887) had higher odds of progressing to diabetes than Class 3. **Conclusions:** Prediabetic individuals fall into three distinct healthy lifestyle latent classes. The distinct associations between the three lifestyle latent classes and glycemic progression to diabetes and regression to normoglycemia support the potential value of lifestyle-based risk stratification in prediabetes management, pending validation in prospective cohorts.

## 1. Introduction

Prediabetes is defined as glucose or Hemoglobin A1c (HbA1c) levels that do not meet the criteria for type 2 diabetes mellitus but are intermediate between normoglycemia and diabetes [1]. In 2021, epidemiological data revealed that the global prevalence of prediabetes had reached unprecedented levels, affecting approximately 464 million individuals worldwide [2]. Within the Chinese population, the cumulative incidence of prediabetes was higher than that observed in other populations [3]. Epidemiological data demonstrated a persistent year-over-year increase in the prevalence of prediabetes, reflecting a clear upward trend [4]. This high-prevalence disease is likely to pose a substantial challenge to healthcare systems.

Current research has demonstrated that individuals with prediabetes are at an elevated risk of progressing to diabetes [5]. Moreover, even if prediabetes does not progress to diabetes, it can still lead to a multitude of adverse health outcomes, including cardiovascular disease (CVD) [6,7] and cognitive impairment [8,9]. Therefore, restoring normoglycemia is broadly recommended for individuals with prediabetes, supported by robust clinical consensus [1]. Notably, evidence demonstrated that this condition can be reversed through targeted lifestyle modifications or pharmacological therapies [6]. A cohort study revealed that over a 6.5-year follow-up, 9% of elderly individuals with prediabetes (HbA1c 5.7–6.4%) developed diabetes, while 13% regressed to normoglycemia [10]. These results indicated that reversal of prediabetes is achievable in elderly populations, underscoring the need for further research to increase reversal rates.

The association between modifiable healthy lifestyle factors and prediabetes reversal centers on several key aspects [1,6,11,12,13]. These include physical activity, dietary patterns, weight control, and blood pressure, which collectively shape glycemic transitions. Cohort data indicate that combining regular exercise, nutrition, and healthy weight maintenance is linked to a reduced risk of diabetes progression and improved glycemic normalization [6]. Yang [14] further demonstrated a positive correlation between weight reduction and the likelihood of achieving normoglycemic reversion. Hypertension has been identified as an independent risk factor for diabetes [15], and clinical guidelines strongly advise maintaining normal blood pressure [11]. However, detrimental lifestyle habits hinder glycemic recovery. Mostafa [16] found that shorter sleep duration may adversely affect glycemic control in prediabetes. Additionally, epidemiological studies indicate that smoking and alcohol consumption hinder prediabetes remission [6].

Extensive research has yielded clear evidence that modifiable healthy lifestyle factors influence glycemic trajectories in prediabetes. However, it is well-established that multiple modifiable risk factors tend to cluster within individuals [17]. For example, obesity is frequently comorbid with elevated blood pressure and physical inactivity [18]. Therefore, any intervention that targets a single risk factor is unlikely to succeed unless it also addresses the clustering dynamics and interactions among multiple healthy lifestyle factors. LCA is a model-based statistical classification technique that aims to classify individuals in a heterogeneous population into homogeneous latent subgroups. The model uses the distribution patterns of observed variables and, through maximum likelihood estimation, identifies the most likely response patterns, thereby classifying individuals into distinct groups. Therefore, compared with other common clustering methods, it is more suitable for capturing the characteristics of lifestyle risk factors [19]. Therefore, compared with traditional clustering analyses, which are essentially based on distance or similarity, LCA is better at revealing the aggregation patterns of lifestyle-related risk factors.

However, the effects of healthy lifestyle classes on glycemic control in prediabetes, as well as the demographic and behavioural characteristics of different classes, remain unclear. Xu [20] conducted a cohort study to systematically evaluate lifestyle patterns among prediabetic populations. The findings demonstrated that individuals with a low-risk healthy lifestyle pattern had a lower risk of diabetes-related adverse outcomes, including death and microvascular complications. However, the only available study investigating the impact of prediabetes-related healthy lifestyle classification on complications categorized identified lifestyle factors through a value-simplistic assignment approach. This methodology overlooked potential complex interactions and synergistic effects among distinct healthy lifestyle components, while also neglecting the influence of holistic lifestyle patterns.

Therefore, our study is based on data from the CHARLS, using LCA to cluster elderly Chinese patients with prediabetes according to different healthy lifestyle factors. We explore the healthy lifestyle classes of individuals with prediabetes and examine how membership in different risk classes relates to glycemic progression. This study aims (1) to explore and name the classes of healthy lifestyles among Chinese patients with prediabetes and (2) to estimate the association of distinct healthy lifestyle classes on glycemic progression among individuals with prediabetes.

## 2. Materials and Methods

### 2.1. Study Population

Data were derived from CHARLS, a nationally representative cohort of Chinese adults aged 45 years and older who are followed biennially [21]. A standardized questionnaire was administered by trained volunteers, followed by a nationwide field survey that enrolled approximately 10,000 households and 17,500 individuals across 150 counties/districts and 450 village committees. Participants underwent biennial follow-ups that included repeated baseline assessments, in-round physical examinations, and blood tests every other round. Five waves of CHARLS are available: 2011, 2013, 2015, 2018 and 2020 Details can be found at http://charls.pku.edu.cn/en/About/About_CHARLS.htm (accessed on 15 January 2025). The Institutional Review Board (IRB) approvals were IRB00001052-11015 for the main household survey, including anthropometrics, and IRB00001052-11014 for biomarker collection.

The dataset was downloaded and accessed for analysis in January 2025. The CHARLS dataset is de-identified and publicly available. The authors did not have access to any information that could identify individual participants during or after data collection.

To assess the generalizability of our findings, we recruited 182 prediabetes patients from the Endocrinology Department and Weight-Loss Clinic of a major tertiary hospital in Shanghai as an independent validation cohort. This external validation section was approved by the Ethics Committee of Shanghai University of Traditional Chinese Medicine (Approval No.: 2026-1-20-06, approved on 3 July 2026). Data collection occurred from 3 July to 22 August 2026, and the manuscript for this section was uploaded on 25 August 2026.

### 2.2. Data Collection

**Figure 1:**
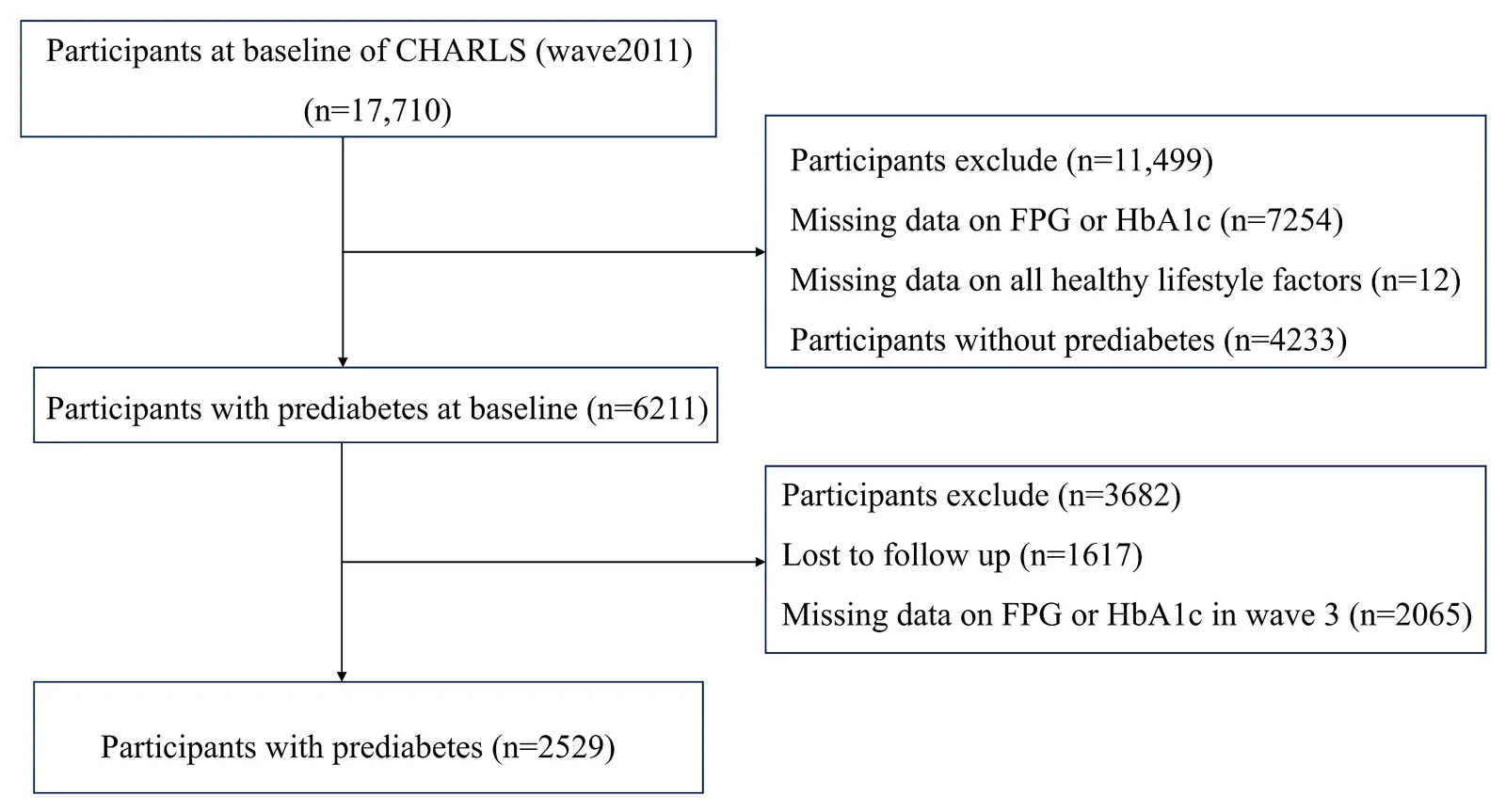
Flow chart of the study participant selection process.

We use the data from Wave 1 and Wave 3, as blood samples were collected only in these waves. Among the 17,710 participants at baseline, exclusions were as follows: individuals without prediabetes at baseline (*n* = 4233); absence of baseline fasting plasma glucose (FPG) or HbA1c measurements (*n* = 7254); missing data on all healthy lifestyle factors (*n* = 12); loss to follow-up (*n* = 1617); and missing either FPG and HbA1c data in Wave 3 (*n* = 2065). Finally, a total of 2529 participants were included in our study (Figure 1).

### 2.3. Measurement

#### 2.3.1. Healthy Lifestyle Factors

Lifestyle factors were operationalized using six components: physical activity, smoking, alcohol consumption, sleep duration, BMI, and blood pressure. These factors were selected based on recommendations from the American Diabetes Association (ADA) and Chinese guidelines for prediabetes management [1,22]. Each variable was dichotomized into healthy and unhealthy categories using predefined cutoffs. Although dichotomizing continuous variables can lead to information loss and may reduce the granularity of risk delineation, the use of clinically meaningful cutoffs was intended to ensure that the identified risk categories could be readily translated into routine clinical practice. Dietary patterns were excluded from the analysis due to the absence of their corresponding data in our dataset. The changes were made by referring to previous studies in the context of the characteristics of the Chinese population [23].

Physical Activity

Healthy physical activity was defined as more than 150 min per week of moderate- to vigorous-intensity activity, including activities such as carrying light loads, regular bicycling, or cleaning tasks. Data were collected using the International Physical Activity Questionnaire—Short Form.

Smoking

We categorized smoking status as current smoker or non-current smoker based on two validated questions: “Have you ever smoked?” and “Do you still have the habit or have you totally quit?”. We defined current smoking as unhealthy category.

Alcohol consumption

Alcohol use was assessed with the question: “Did you drink any alcoholic beverages in the past year?” Non-drinking was considered the healthy category; any alcohol use was considered unhealthy.

Sleep duration

Sleep duration was defined by patients’ self-reported average night sleep time, 6–9 h is defined as healthy category, and others are defined as unhealthy category.

BMI

BMI was calculated as weight in kilograms divided by the square of height in meters. A BMI ≥ 25 kg/m^2^ was categorized as unhealthy.

High Blood Pressure

Hypertension was defined as systolic blood pressure ≥140 mmHg or diastolic blood pressure ≥ 90 mmHg. Height, weight, and BP data were measured by trained professionals.

#### 2.3.2. Assessment of Prediabetes Transition

Glycemic status was defined according to the ADA criteria [1]. Diabetes was defined as HbA1c ≥ 6.5% (48 mmol/mol), FPG ≥ 126 mg/dL, or self-reported diabetes. Prediabetes was HbA1c 5.7–6.4% (39–46 mmol/mol) or FPG 100–125 mg/dL. Normoglycemia was defined as FPG <100 mg/dL and HbA1c < 5.7% (39 mmol/mol).

According to the diagnostic criteria proposed, patients with prediabetes are divided into three groups, (1) prediabetes to normoglycemia; (2) prediabetes to prediabetes; (3) prediabetes to diabetes.

#### 2.3.3. Covariates

Self-reported sociodemographic variables included age, gender, ethnicity, marital status, educational status (primary school or below, middle school, and high school or above), residence (urban, rural), employment status (yes, no), self-reported dyslipidaemia (yes, no), self-reported cardiovascular disease (CVD) (yes, no), cognition (Normal, Mild Impairment, Moderate Impairment, Severe Impairment) and Waist-to-Height Ratio (WtHR).

According to the Mini-Mental State Examination, participants with scores of 27–30 were classified as cognitively normal, while those scoring below 27 were identified as having cognitive impairment and further stratified into mild (21–26), moderate (10–20), and severe (0–9) severity grades.

WtHR is calculated as waist circumference (WC) divided by height, with both measurements obtained in the same units. Although BMI and WtHR are both anthropometric indicators, the information they reflect in clinical pathophysiology differs. BMI mainly reflects global obesity or overall nutritional status, whereas WtHR more specifically points to central obesity and visceral fat accumulation, which is closely related to glucose metabolism—a fact that underlies our choice to include it as a covariate. All anthropometric measurements were obtained by trained and certified research staff following standardized protocols.

### 2.4. Statistical Analysis

Normality of continuous variables was assessed using the one-sample Kolmogorov–Smirnov test. Non-normally distributed variables were described by median with interquartile range (M [P25, P75]) and analysed with the Mann-Whitney U test. Categorical variables were summarized as frequency (*n*, %) and compared using chi-square test. Descriptive statistics described the baseline prevalence and changes in blood glucose. Variables with significant baseline differences informed covariate selection for subsequent models.

We used LCA to identify subgroups of prediabetes based on six healthy lifestyle factors. The optimal number of classes was determined primarily by lower values of the Akaike information criterion (AIC), Bayesian information criterion (BIC), and the sample-size-adjusted BIC (aBIC), together with the clinical interpretability of the classes. Additionally, higher entropy and significant results from the Vuong–Lo–Mendell–Rubin (VLMR) likelihood ratio test were considered as secondary criteria to ensure clear distinctions between the classes. We performed external validation using LCA. The chi-square test and Kruskal–Wallis test were used to compare baseline characteristics across healthy lifestyle groups.

We conducted multinomial logistic regression to assess associations between healthy lifestyle classes and glycemic transitions in prediabetes. The model adjusted for age, gender, place of residence, marital status, educational level, employment status, self-reported dyslipidaemia, self-reported CVD, cognition and WtHR. Missing covariate data were handled with multiple imputation. Additionally, as a sensitivity analysis, we repeated our analysis using the original, unimputed dataset. All statistical analyses were performed using SPSS 26.0 and Mplus 8.3. A two-sided *p*-value of <0.05 was considered statistically significant.

## 3. Results

### 3.1. Participant Characteristics

We depict the four-year blood glucose transition patterns (Figure S1). Among participants with prediabetes at baseline, 1644 individuals (65%) remained in prediabetes, 322 (13%) progressed to diabetes, and 563 (22%) reverted to normoglycemia.

Baseline characteristics are presented (Table S1). Among the 2925 participants, the median age was 59 years; 1136 (45.2%) participants were male; 2364 (93.5%) participants were of Han ethnicity; 2099 (83.0%) resided in urban areas. Significant differences (*p* < 0.05) across glycemic progression were observed for age, gender, ethnicity, education, employment status, self-reported hypertension, dyslipidaemia, and serum biomarkers.

### 3.2. Latent Classes Based on Six Healthy Lifestyle Factors

We clustered healthy lifestyles into three categories (Table S2). During latent class enumeration, information criteria (AIC, BIC, aBIC) consistently exhibited declined from one-class to three-class solutions, with an inflection point at the four-class model where the value rose. The bootstrap likelihood ratio test (BLRT) was statistically significant only for the four-class model (*p* < 0.001). However, the absolute improvement in fit indices from three classes to four classes was very small (ΔAIC = −19.2, ΔaBIC = −0.62), suggesting that adding the fourth class yields limited additional improvement. This pattern suggests that, although statistically distinguishable, the incremental improvement in model fit with additional classes diminishes substantially beyond three classes, necessitating careful evaluation of clinical interpretability and parsimony in selecting the optimal solution. Notably, the three-class model exhibited significantly higher entropy values than the four-class model (Δentropy = −0.15), reflecting superior classification performance. Additionally, the four-class model demonstrated limited clinical translatability (Table S4). In the four-class model, the conditional probabilities for Class 3 across all indicators hover around 0.40–0.50, indicating low clinical discriminability; extreme features are observed only for alcohol use, which overlaps with the features of Class 4, thereby reducing its clinical utility. Moreover, we report the posterior probabilities for the three-class model (Table S3), which are robust. Consequently, the three-class model was retained based on parsimony and actionable relevance.

**Figure 2:**
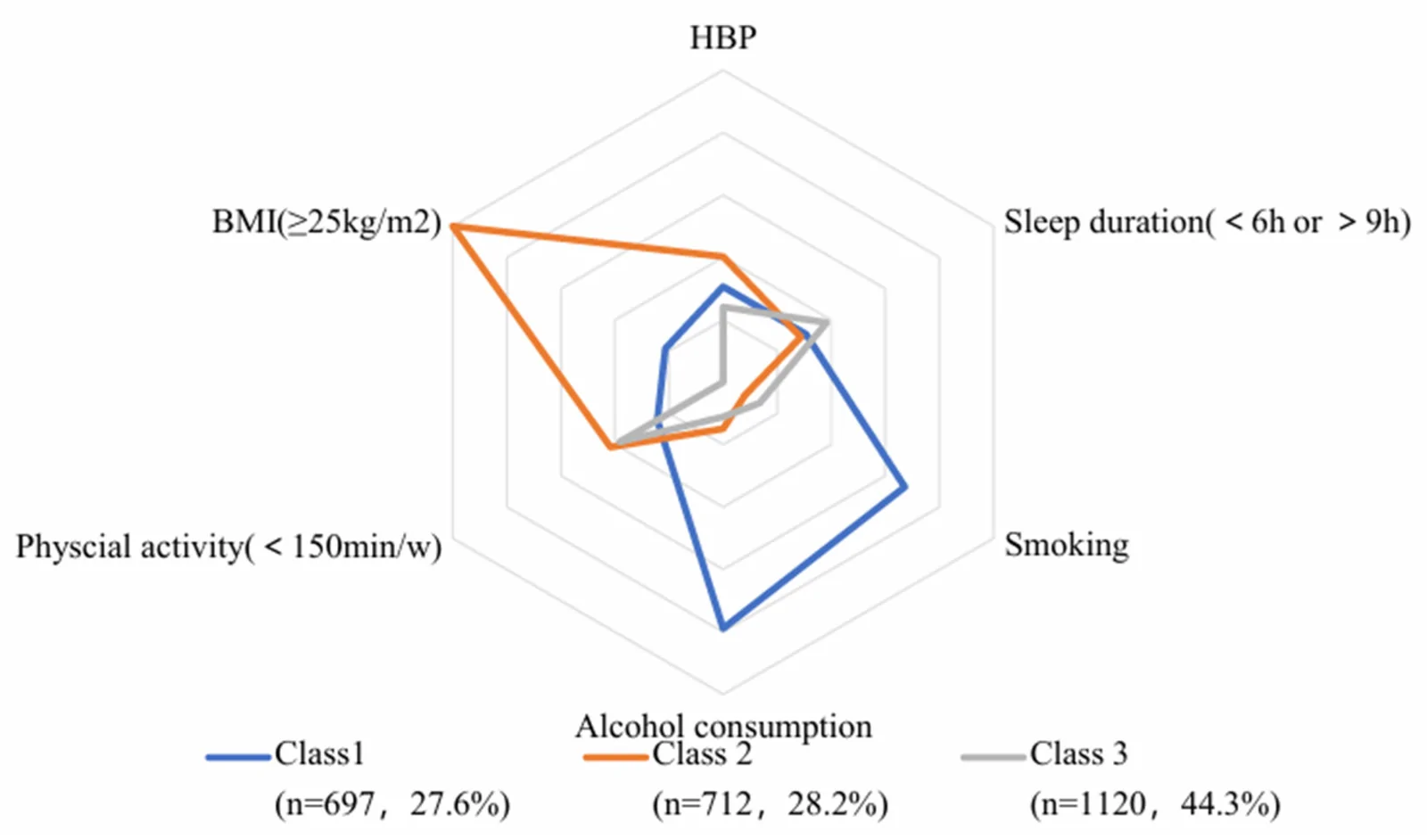
Probabilities of healthy lifestyle across latent classes. Abbreviations: HBP, high blood pressure; BMI, body mass index. Class 1 = smoking and drinking group; Class 2 = high-BMI group; Class 3 = healthy lifestyle group. The radial distance from the chart center reflects the degree of deviation from recommended health guidelines, with larger distances indicating more pronounced behavioural risk factors.

We present the conditional probabilities (Table S5). Based on the conditional probability distributions of the items, we identified three latent classes. Participants in Class 1 (*n* = 697, 27.6%) had relatively 21% high BMI, 30% high blood pressure, 79% reported drinking, and 67% reported smoking, so we call them “smoking and drinking group”. Class 2 (*n* = 712, 28.2%) exhibited a higher BMI level, with less unhealthy lifestyle percentages, designated the “high-BMI group”. Class 3 (*n* = 1120, 44.3%) exhibited high endorsement probabilities across healthy lifestyle factors, designated the “healthy lifestyle group”. We present a radar chart (Figure 2).

### 3.3. Baseline Characteristics of Healthy Lifestyle Classes

Univariable analyses were conducted to compare sociodemographic variables across healthy lifestyle classes. There were significant differences in age, gender, residence, marriage, education, and employment status (Table 1). Pairwise comparisons showed that, compared with Class 3, Class 1 had a higher proportion of males, higher education levels, and a greater proportion of employed individuals. Moreover, Class 2 exhibited a lower proportion of rural residents and a younger age distribution, whereas Class 3 displayed lower rates of marriage and cohabitation. Univariable analyses of health outcomes revealed statistically significant differences in dyslipidaemia, CVD, cognitive function, depression, and in lipid and glycemic indicators (*p* < 0.05).

**Figure 3:**
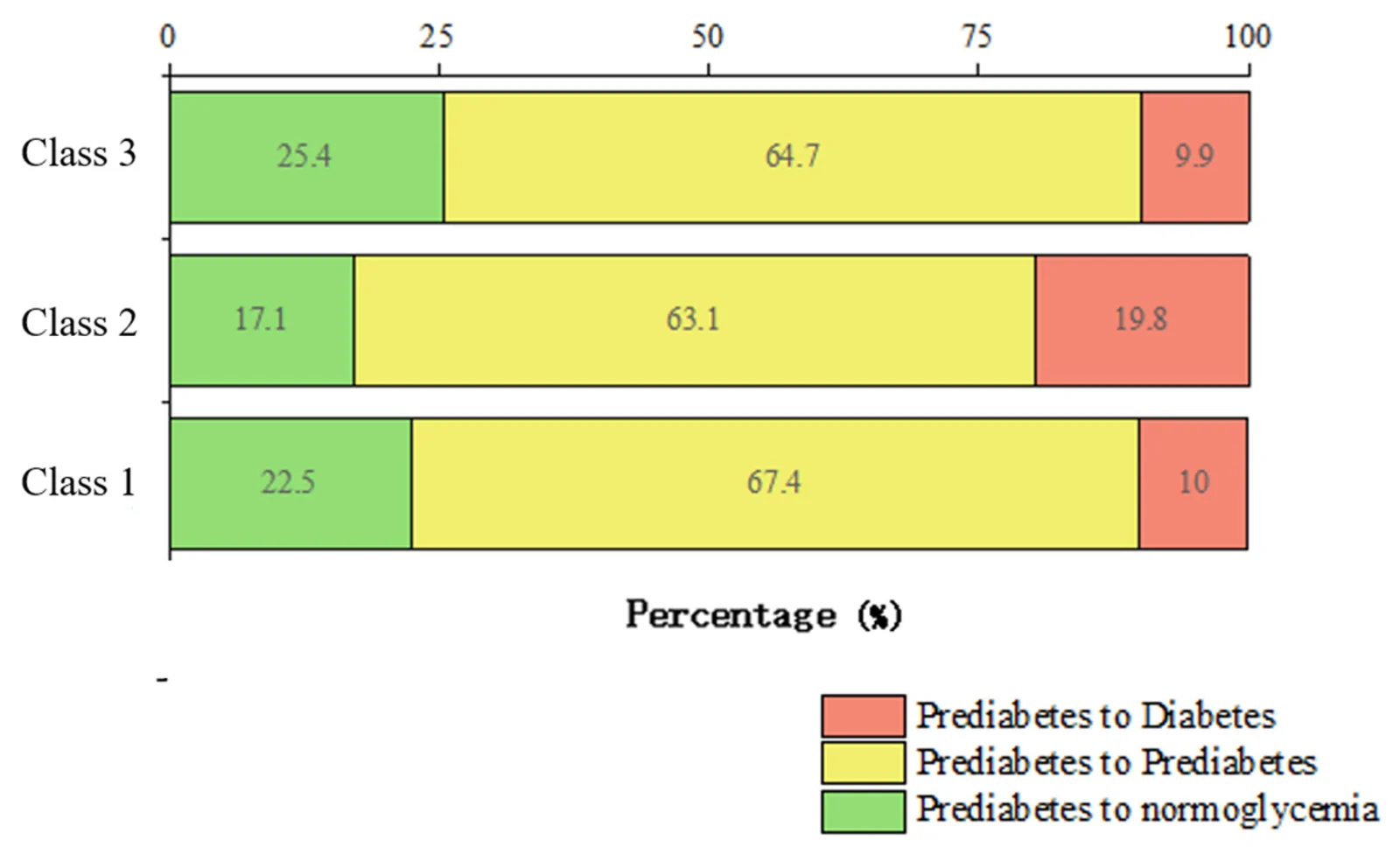
Changes in blood glucose in prediabetic patients among different healthy lifestyle classes. Class 1 = smoking and drinking group; Class 2 = high-BMI group; Class 3 = healthy lifestyle group. The percentage bar charts depict four-year blood glucose change percentages among participants across three healthy lifestyle classes, utilizing data from two survey waves (2011 and 2015).

We demonstrated glycemic transitions across healthy lifestyle classes (Figure 3). In Class 2, 17.1% of patients regressed to normoglycemia and 19.8% progressed to diabetes. Class 1 had the highest regression rate (22.5%) and lowest progression rate (10%), while Class 3 exhibited 25.4% regression and 9.9% progression.

### 3.4. Association Between Health Risk Classes and Glycemic Status Transitions

Keeping prediabetes as the reference group, multinomial logistic regression analysis was conducted to explore the associations between healthy lifestyle classes and glycemic transitions (Table 2). According to the unadjusted model, Class 2 had lower odds of remaining in the prediabetic state (OR, 0.694; 95% CI, 0.544 to 0.884) and higher odds of progressing to diabetes (OR, 2.051; 95% CI, 1.588 to 2.701).

After adjusting for age, gender, place of residence, marital status, educational level, employment status, presence of dyslipidaemia, presence of CVD, cognition and WtHR, Class 1 had lower odds of returning to normal glycemia (OR, 0.736; 95% CI, 0.554 to 0.978). Additionally, Class 2 showed bidirectional risks: higher odds of both remaining prediabetic (OR, 0.706; 95% CI, 0.526 to 0.947) and progressing to diabetes (OR, 2.394; 95% CI, 1.745 to 3.286). Sensitivity analysis yielded comparable results (Table S7). Furthermore, separate analyses conducted for each healthy lifestyle factor (Table S6) revealed that only BMI demonstrated a significant difference in glycemic reversion.

### 3.5. External Validation Results

To evaluate the generalizability of the main analysis results, we included 182 prediabetes patients as an independent external validation cohort. Compared with the CHARLS-based main study cohort, this validation cohort tended to be younger, predominantly urban residents, and had higher educational attainment (Table S8).

We applied the same LCA workflow as in the main analysis and fitted models with 1–5 classes sequentially (Table S9). Although the two-class model yielded the lowest AIC and BIC values, the validation cohort was small, which substantially reduces the statistical power of the likelihood ratio tests; consequently, the BLRT for the three-class model was not statistically significant (*p* = 0.667), indicating that this test has limited interpretive value in this context. Secondly, the three-class model demonstrated excellent classification quality (entropy = 0.950). Thirdly, and most importantly, the conditional probability distributions for the three classes in the validation cohort were highly concordant with those in the main study (Table S10), providing strong evidence that this three-class classification structure is reproducible across different populations.

## 4. Discussion

We found that among middle-aged and elderly Chinese participants, baseline prediabetes prevalence was 47%. Furthermore, 5% progressed from normoglycemia to diabetes, considerably lower than the 13% who progressed from prediabetes to diabetes. These results are consistent with prior research [10]. Prediabetic status was associated with a significantly higher risk of incident diabetes than normal glycemia.

Our study categorized the healthy lifestyle of prediabetic individuals into three different classes base on six factors. Subsequently, the class labels were refined through consensus-building to ensure clinical relevance. The three classes are the smoking and drinking group, the high-BMI group, and the healthy lifestyle group.

The healthy lifestyle group constitutes the largest portion of the sample, approximately 1120 (44%), and significantly exceeds the other groups in size. However, Zhang’s LCA study [24] found that only 21% of Chinese elderly maintained a healthy lifestyle, a discrepancy that may reflect methodological heterogeneity in assessing healthy lifestyles and population differences. Our study specifically targeted individuals with prediabetes. When glycemic abnormalities are detected, these individuals tend to develop heightened risk perception regarding metabolic consequences [25], as evidenced by greater receptivity to health guidance, a stronger intent to modify lifestyle, and sustained adherence to healthy behaviours. This class also exhibits lower marriage rates, reduced urban residence, and older age. People of older age and retirees tend to pay more attention to their health [26]. These findings may provide a basis for future investigations into tiered and progressive management strategies, including enhanced monitoring and the potential use of pharmacotherapy for individuals with different risk profiles.

The smoking and drinking group accounted for 27.6% of the study population and was characterized by male predominance, higher educational attainment, and full-time employment. Evidence confirms a robust behavioural clustering of smoking and alcohol use [27]. Historically, gender inequality and sociocultural norms relegated Chinese elderly women to lower social status and limited educational opportunities, which discouraged female smoking and drinking [28]. These patterns persist among older generations despite progress. Therefore, future research may investigate the potential value of tailored, context-specific strategies that consider occupational characteristics and stress triggers, incorporate tobacco and alcohol cessation support, evidence-based stress-reduction approaches, workplace-based contextual prompts, and professional health education, with the aim of bridging the gap between health knowledge and behaviour and facilitating the integration of health-promoting behaviours into daily routines.

The high-BMI group accounted for 28.2% and tended to have higher urban residency rates and be younger than the healthy lifestyle group. Urban populations generally engage in lower levels of physical activity compared with their non-urban counterparts [29], a deficit that is compounded by limited access to outdoor recreational spaces in urban environments. This pattern may heighten obesity susceptibility due to reduced activity and energy-dense dietary patterns [30]. Building on our findings, future research may explore the potential value of several weight management support strategies, including (1) personalized exercise prescriptions and dietary protocols; (2) accessible green exercise infrastructure and incentivized physical activity programs; and (3) mobile health platforms and AI-driven behavioural nudges.

Compared with the healthy lifestyle group, the smoking and drinking group and the high-BMI group demonstrated a significantly higher risk of progression to diabetes. In diabetic patients, pancreatic β-cells dedifferentiate into α-cells, disrupting glucose homeostasis by impairing insulin secretion. Insulin secretory capacity inversely correlates with the extent of dedifferentiation, with evidence suggesting this process serves as an adaptive mechanism to evade apoptosis under metabolic stress while retaining potential for redifferentiation upon environmental amelioration [31]. Therefore, for prediabetic individuals, strategies targeting insulin resistance mitigation and β-cell functional restoration could synergistically promote glycemic normalization. Within this study, the cluster of risk characteristics observed in the high-risk group is highly concordant with the known pathophysiological pathways by which ectopic lipid deposition mediates dysglycemia. This association suggests that evidence-based weight reduction and physical activity interventions may hold substantial potential utility for this subgroup. Even among individuals with a normal BMI, smoking impairs β-cell function, and alcohol inhibits insulin secretion. Concurrent smoking and alcohol use synergistically exacerbate metabolic burden through overlapping pathways (e.g., oxidative stress, inflammation, lipid dysregulation), with epidemiological studies demonstrating the highest risk of β-cell impairment in dual users [32]. Our findings are in line with previous evidence suggesting that sustained lifestyle modification, including weight management, physical activity promotion, and reduction of smoking and alcohol exposure, may play an important role in improving glycemic control and facilitating diabetes remission among individuals with prediabetes.

Compared with the healthy lifestyle group, the high-BMI group had a significantly greater risk of progression to diabetes. The detrimental effects of obesity and adipose tissue on glycemic regulation are well established [33]. Obesity exacerbates cellular senescence, driving insulin resistance and impairing tissue regeneration. Additionally, skeletal muscle plays a critical role in regulating systemic blood glucose homeostasis. Among 203,767 individuals tracked for three years, Hong [34] revealed an inverse association between relative muscle mass and diabetes incidence. Mechanistically, skeletal muscle is the major site of insulin-stimulated glucose disposal, accounting for approximately 80–90% of glucose uptake under normal physiological conditions [35]. Consequently, diminished skeletal muscle mass directly impairs systemic glucose disposal capacity. These findings suggest that future interventions for individuals in the high-BMI group could explore the potential benefits of structured exercise programs, including resistance training, in promoting favorable changes in body composition through improvements in muscle mass and reductions in fat mass.

Compared with the healthy lifestyle group, the smoking and drinking group demonstrated no statistically significant association with progression from prediabetes to diabetes. Although prior studies have proved that smoking and drinking accelerate β-cell exhaustion [32], our study failed to detect a significant effect, possibly because the follow-up period was only four years. Glycemic variability has a multifactorial basis, and focusing solely on smoking and alcohol use inadequately explains its determinants. Unmeasured dietary confounders, genetic polymorphisms, and therapeutic interventions may modulate these relationships in different ways. Given the well-established health benefits of prediabetes reversal, lifestyle modifications in individuals from the smoking and drinking group should be complemented by prioritizing smoking and alcohol cessation counselling.

Notably, when individual risk factors were examined separately, only BMI showed a significant association with glycemic reversion. This finding stands in stark contrast to the results from the LCA and strongly suggests that traditional univariable analyses may substantially underestimate the joint effects of healthy lifestyles, while the specific combinatorial patterns of multiple risk factors exert a far greater influence on glycemic transitions than any single factor.

Our study found clear and clinically meaningful lifestyle cluster patterns in prediabetes populations, and these clusters were significantly associated with glycemic status. These findings provide a foundation for future longitudinal research—by tracking behavioural trajectories at different stages of glycemic progression and by combining repeated measurements of lifestyle behaviours with dynamic monitoring of glycemic status, researchers can further explore how transitions in behavioural patterns (e.g., moving from an unhealthy cluster to a healthy cluster) influence changes in blood glucose. Such studies can not only strengthen causal inference but also clarify the optimal timing and targets for lifestyle interventions. Moreover, our study only analysed changes in blood glucose over a four-year period. Future studies with longer follow-up would capture more cases of disease progression and remission.

### Limitation

There are several limitations in our research. Firstly, though our study utilized a large-scale database, the data did not comprehensively capture all relevant variables, such as medical history and dietary data. Nevertheless, we included BMI and blood pressure as downstream biological markers of long-term dietary patterns, which can partially reflect the cumulative metabolic effects of diet. Additionally, our main findings suggest clustering among factors such as insufficient physical activity, sleep disturbances, and tobacco and alcohol exposure, which are associated with diabetes risk. While these results provide valuable insights, future research should incorporate dietary assessments to validate and deepen these findings. Secondly, there was a relatively high proportion of missing blood glucose data among the participants. Although CHARLS collected blood samples, in large community surveys, some respondents were unwilling or unable to undergo blood drawing, and some samples did not pass laboratory quality control, leading to incomplete biomarker coverage. Previous CHARLS-based studies have faced similar issues [36]. If individuals with missing glucose data differ systematically from those with complete data in age, health status, or lifestyle, selection bias may be introduced, thereby affecting the reliability and generalizability of our findings. Thirdly, the study sample was predominantly Chinese due to database limitations, potentially limiting the applicability of the findings given the cultural disparities in healthy behaviours. Although the external validation supported the robustness of the lifestyle cluster approach, the validation was still limited to Chinese populations, and the applicability of this typology framework across different cultural contexts will require cross-national and cross-ethnic studies to validate. Nevertheless, our core findings align mechanistically with prior study, while prospective validation in migrant populations with longitudinal geospatial tracking data remains imperative.

## 5. Conclusions

This study is based on longitudinal cohort data from 2529 Chinese adults with prediabetes and used LCA to identify three distinct lifestyle latent classes—the smoking and drinking group, the high-BMI group, and the healthy lifestyle group. These clusters exhibited differential associations with glycemic transitions: the smoking and drinking group carried a higher risk of progression to diabetes, while the high-BMI group not only accelerated disease progression but also significantly hindered the reversal of glycemia to normal. These findings indicate that unhealthy lifestyle factors in prediabetes tend to cluster in specific patterns rather than occur in isolation, and the combined lifestyle phenotype has a stronger influence on bidirectional glycemic transitions than single lifestyle factors. Future intervention studies could explore whether lifestyle-cluster–based strategies can improve the effectiveness of diabetes prevention by targeting individuals with specific behavioural patterns, thereby optimizing the timing and content of lifestyle interventions during the prediabetic stage.

## Figures and Tables

**Table 1:** Baseline characteristics of healthy lifestyle classes.

Covariates	Latent Class			*p*
	Class 1 ^a^ (*n* = 697, 27.6%)	Class 2 ^a^ (*n* = 712, 28.2%)	Class 3 ^a^ (*n* = 1120, 44.3%)	
Age	59 (54, 65) ^b^	57 (51, 62) ^b,c^	60 (54, 67) ^c^	**<0.001**
Gender				**<0.001**
Male	582 (84.2) ^b,c^	205 (29.0) ^b^	349 (31.2) ^c^	
Female	109 (15.8)	503 (71.0)	768 (68.8)	
Ethnic group				0.592
Han	651 (93.4)	671 (94.2)	1042 (93.0)	
Others	46 (6.6)	41 (5.8)	78 (7.0)	
Residence				**0.016**
Rural	588 (84.4)	566 (79.6) ^b^	945 (84.4) ^b^	
Urban	109 (15.6)	145 (20.4)	175 (15.6)	
Marriage				**0.001**
Married and living with spouse	607 (87.1) ^b^	626 (87.9) ^c^	919 (82.1) ^b,c^	
Others	90 (12.9)	86 (12.1)	201 (17.9)	
Education				**<0.001**
Primary school below	251 (36.0) ^b^	351 (49.3) ^b^	604 (54.0) ^c^	
Primary school	185 (26.5)	150 (21.1)	238 (21.3)	
Middle school or above	261 (37.5)	211 (29.6)	277 (24.8)	
Employment Status				**<0.001**
Working	564 (81.0) ^b,c^	442 (62.3) ^b^	728 (65.0) ^c^	
Not working	132 (19.0)	268 (37.7)	392 (35.0)	
Chronic diseases				
Dyslipidaemia				**<0.001**
Yes	312 (7.6) ^b^	768 (18.5) ^b,c^	528 (8.0) ^c^	
No	3792 (92.4)	3378 (81.5)	6048 (92.0)	
CVD				**<0.001**
Yes	66 (9.6) ^b^	123 (17.3) ^b,c^	147 (13.2) ^c^	
No	624 (90.4)	587 (82.7)	965 (86.8)	
CKD				0.131
Yes	44 (6.4)	32 (4.5)	75 (6.8)	
No	646 (93.6)	677 (95.5)	1035 (93.2)	
Cognition				**<0.001**
Normal	150 (21.5)	131 (18.4) ^b^	358 (32.0) ^b^	
Mild Impairment	448 (64.3)	494 (69.4)	645 (57.6)	
Moderate Impairment	95 (13.6)	84 (11.8)	115 (10.3)	
Severe Impairment	4 (0.6)	3 (0.4)	1 (0.1)	
Depression (CES-D-10 score)	7 (3, 12) ^b^	7 (4, 12)	8 (4, 12) ^b^	**0.015**
Grip strength (kg)	35 (18, 42) ^b,c^	30 (24, 36) ^b,d^	28 (23, 34) ^c,d^	**<0.001**
Waist circumference (cm)	82 (77, 89) ^b,c^	95 (90, 100) ^b,d^	81 (76, 86) ^c,d^	**<0.001**
WtHR	0.51 (0.48, 0.55) ^b^	0.60 (0.57, 0.64) ^b,c^	0.52 (0.49, 0.55) ^c^	**<0.001**
TC (mg/dL)	191.0 (168.6, 215.3)	199.1 (173.4, 225.4)	193.3 (170.10, 216.9)	**0.002**
LDL-c (mg/dL)	112.5 (92.8, 134.9) ^b,c^	121.8 (98.6, 147.7) ^c^	118.9 (97.2, 139.0) ^b^	**<0.001**
HDL-c (mg/dL)	112.5 (92.8, 134.9) ^b^	121.8 (98.6, 147.7) ^b,c^	118.9 (97.2, 139.0) ^c^	**<0.001**
FPG (mg/dL)	107 (103, 113) ^b^	107 (103, 113) ^c^	106 (102, 111) ^b,c^	**<0.001**
HbA1c (%)	5.1 (4.9, 5.4) ^b^	5.2 (5.0, 5.5) ^b,c^	5.2 (4.9, 5.4) ^c^	**<0.001**

Note: ^a^ Class 1 = smoking and drinking group; Class 2 = high-BMI group; Class 3 = healthy lifestyle group. ^b,c,d^ denote statistically significant differences between pairwise comparisons. WtHR, Waist-to-Height Ratio; TC, total cholesterol; LDL-c, low-density lipoprotein–cholesterol; HDL-c, high-density lipoprotein–cholesterol; CES-D-10, 10-item Center for Epidemiologic Studies Depression Scale; CKD, Chronic Kidney Disease. Bold represents all statistically significant *p*-values (*p* < 0.05).

**Table 2:** Multivariable associations of change in blood glucose with healthy lifestyle classes.

	Unadjusted Model				Adjusted Model ^a^			
	Prediabetes to Normoglycemia		Prediabetes to Diabetes		Prediabetes to Normoglycemia		Prediabetes to Diabetes	
	OR	*p*	OR	*p*	OR	*p*	OR	*p*
Class 1 ^b^	0.853 (0.680, 1.070)	0.169	0.973 (0.706, 1.341)	0.866	0.736 (0.554, 0.978)	**0.033**	1.115 (0.750, 1.659)	0.564
Class 2 ^b^	0.694 (0.544, 0.884)	**0.00** **3**	2.051 (1.588, 2.701)	**<0.001**	0.706 (0.526, 0.947)	**0.020**	2.394 (1.745, 3.286)	**<0.001**
Class 3 ^b^	1 (Ref.)		1 (Ref.)		1 (Ref.)		1 (Ref.)	

Note: ^a^ Adjusted for age, gender, place of residence, marital status, educational level, working, presence of dyslipidaemia, presence of CVD, cognition and WtHR. ^b^ Class 1 = smoking and drinking group; Class 2 = high-BMI group; Class 3 = healthy lifestyle group. Multiple imputation was used for missing covariates, under the assumption that data were Missing at Random. OR, odds ratio. Bold represents all statistically significant *p*-values (*p* < 0.05).

## Data Availability

The data presented in this study are available in CHARLS at http://charls.pku.edu.cn/en/About/About_CHARLS.htm. These data were derived from the following resources available in the public domain: http://charls.pku.edu.cn/en/About/About_CHARLS.htm (accessed on 15 January 2025).

## Supplementary Materials

The following supporting information can be downloaded at: https://www.mdpi.com/article/10.3390/healthcare14182966/s1, Table S1. Baseline characteristics of the study participants according to glycemic condition. Table S2. Fit statistics models from one to five classes. Table S3. Average latent class probabilities for most likely latent class membership by latent class (column). Table S4. The latent-class–conditional probability distributions for the four-class model. Table S5. The latent-class–conditional probability distributions for the three-class model. Table S6. Association between healthy lifestyle factors and transition of glycemic Status. Table S7. Sensitivity analysis of the association between healthy lifestyle classes and glycemic change. Table S8. Baseline characteristics of 182 individuals with prediabetes. Table S9. Fit statistics models from one to five classes of 182 individuals with prediabetes. Table S10. The latent-class–conditional probability distributions of 182 individuals with prediabetes. Figure S1. Blood glucose transition patterns in CHARLS in the 4-year period. Figure S2. Data Missingness Pattern.

## Abbreviations

The following abbreviations are used in this manuscript:

OR	Odds Ratio
BMI	Body Mass Index
HbA1c	Haemoglobin A1c
FPG	Fasting Plasma Glucose
CHARLS	China Health and Retirement Longitudinal Study
IBR	Institutional Review Board
AIC	Akaike Information Criterion
BIC	Bayesian Information Criterion
aBIC	Sample-size-adjusted BIC
ADA	American Diabetes Association
WtHR	Waist-to-Height Ratio
CVD	Cardiovascular Disease
CKD	Chronic Kidney Disease
LCA	Latent Class Analysis
HBP	High Blood Pressure
TC	Total Cholesterol
LDL-c	Low-Density Lipoprotein–cholesterol
HDL-c	High-Density Lipoprotein–cholesterol
CES-D-10	10-item Center for Epidemiologic Studies Depression Scale
VLMR	Vuong–Lo–Mendell–Rubin
BLRT	Bootstrap Likelihood Ratio Test
WC	Waist Circumference

## References

[1] American Diabetes Association Professional Practice Committee (2023). 2. Diagnosis and Classification of Diabetes: Standards of Care in Diabetes—2024. Diabetes Care.

[2] Rooney M.R., Fang M., Ogurtsova K., Ozkan B., Echouffo-Tcheugui J.B., Boyko E.J., Magliano D.J., Selvin E. (2023). Global Prevalence of Prediabetes. Diabetes Care.

[3] Li Y., Teng D., Shi X., Qin G., Qin Y., Quan H., Shi B., Sun H., Ba J., Chen B. (2020). Prevalence of diabetes recorded in mainland China using 2018 diagnostic criteria from the American Diabetes Association: National cross sectional study. BMJ.

[4] Xia M., Liu K., Feng J., Zheng Z., Xie X. (2021). Prevalence and Risk Factors of Type 2 Diabetes and Prediabetes Among 53,288 Middle-Aged and Elderly Adults in China: A Cross-Sectional Study. Diabetes Metab. Syndr. Obes..

[5] Tabák A.G., Herder C., Rathmann W., Brunner E.J., Kivimäki M. (2012). Prediabetes: A high-risk state for diabetes development. Lancet.

[6] Cao Z., Li W., Wen C.P., Li S., Chen C., Jia Q., Li W., Zhang W., Tu H., Wu X. (2023). Risk of Death Associated with Reversion from Prediabetes to Normoglycemia and the Role of Modifiable Risk Factors. JAMA Netw. Open.

[7] Huang J.Y., Tse Y.K., Li H.L., Chen C., Zhao C.T., Liu M.Y., Wu M.Z., Ren Q.W., Yu S.Y., Hung D. (2023). Prediabetes Is Associated with Increased Risk of Heart Failure Among Patients with Atrial Fibrillation. Diabetes Care.

[8] Casagrande S.S., Lee C., Stoeckel L.E., Menke A., Cowie C.C. (2021). Cognitive function among older adults with diabetes and prediabetes, NHANES 2011-2014. Diabetes Res. Clin. Pract..

[9] Xing X., Wang Y., Shen Z., Pan F., Cai G. (2024). Association of prediabetes progression and regression with cognitive decline: Findings from the CHARLS. Diabet. Med..

[10] Rooney M.R., Rawlings A.M., Pankow J.S., Echouffo Tcheugui J.B., Coresh J., Sharrett A.R., Selvin E. (2021). Risk of Progression to Diabetes Among Older Adults with Prediabetes. JAMA Intern. Med..

[11] Chinese Society of Endocrinology, Chinese Diabetes Society, Chinese Endocrinologist Association (2023). Intervention for adults with pre-diabetes: A Chinese expert consensus (2023 edition). Chin. J. Diabetes.

[12] Kontochristopoulou A.M., Karatzi K., Karaglani E., Cardon G., Kivelä J., Wikström K., Iotova V., Tsochev K., Tankova T., Rurik I. (2022). Sociodemographic, anthropometric, and lifestyle correlates of prediabetes and type 2 diabetes in europe: The Feel4Diabetes study. Nutr. Metab. Cardiovasc. Dis..

[13] O’Donoghue G., O’Sullivan C., Corridan I., Daly J., Finn R., Melvin K., Peiris C. (2021). Lifestyle Interventions to Improve Glycemic Control in Adults with Type 2 Diabetes Living in Low-and-Middle Income Countries: A Systematic Review and Meta-Analysis of Randomized Controlled Trials (RCTs). Int. J. Environ. Res. Public Health.

[14] Yang H., Zhang M., Nie J., Zhang M., Lu G., Chen R., He Q. (2023). Associations of obesity-related indices with prediabetes regression to normoglycemia among Chinese middle-aged and older adults: A prospective study. Front. Nutr..

[15] Li Y., Jiang Y., Lin J., Wang D., Wang C., Wang F. (2022). Prevalence and associated factors of diabetes mellitus among individuals aged 18 years and above in Xiaoshan District, China, 2018: A community-based cross-sectional study. BMJ Open.

[16] Mostafa S.A., Mena S.C., Antza C., Balanos G., Nirantharakumar K., Tahrani A.A. (2022). Sleep behaviours and associated habits and the progression of pre-diabetes to type 2 diabetes mellitus in adults: A systematic review and meta-analysis. Diabetes Vasc. Dis. Res..

[17] Noble N., Paul C., Turon H., Oldmeadow C. (2015). Which modifiable health risk behaviours are related? A systematic review of the clustering of Smoking, Nutrition, Alcohol and Physical activity (‘SNAP’) health risk factors. Prev. Med..

[18] Papassotiriou I., Spiliopoulou S., Dragonas D., Tsoutsoura N., Korompoki E., Manios E. (2025). The relation between body mass index and target organ damage and the mediating role of blood pressure. Eur. J. Clin. Nutr..

[19] Graf S., Cecchini M. (2018). Identifying patterns of unhealthy diet and physical activity in four countries of the Americas: A latent class analysis. Rev. Panam. Salud Publica.

[20] Xu X., Li J., Yu Y., Tan X., Xu F., Wang B., Wang N., Lu Y. (2024). Association of combined healthy lifestyle with risk of adverse outcomes in patients with prediabetes. Diabetes Metab. Res. Rev..

[21] Zhao Y., Hu Y., Smith J.P., Strauss J., Yang G. (2014). Cohort profile: The China Health and Retirement Longitudinal Study (CHARLS). Int. J. Epidemiol..

[22] American Diabetes Association (2020). 3. Prevention or Delay of Type 2 Diabetes: Standards of Medical Care in Diabetes—2021. Diabetes Care.

[23] Chen W., Wang X., Chen J., You C., Ma L., Zhang W., Li D. (2023). Household air pollution, adherence to a healthy lifestyle, and risk of cardiometabolic multimorbidity: Results from the China health and retirement longitudinal study. Sci. Total Environ..

[24] Zhang L., Bi X., Ding Z. (2021). Health lifestyles and Chinese oldest-old’s subjective well-being-evidence from a latent class analysis. BMC Geriatr..

[25] Yildirim Duman J.G. (2021). Self-Management of Chronic Diseases: A Descriptive Phenomenological Study. Soc. Work Public Health.

[26] Wang B., Lu J. (2024). Life Chances, Subjective Perceptions, and Healthy Lifestyles in Older Adults: Longitudinal Evidence from China. J. Gerontol. Ser. B Psychol. Sci. Soc. Sci..

[27] Lee Y.-H., Kumwiang K., Chiang T., Shelley M., Chang Y.-C. (2024). Twenty-Year Trends and Urban–Rural Disparities in Smoking, Alcohol Consumption, and Dual Consumption Among Chinese Older Adults. Int. J. Ment. Health Addict..

[28] Li Y., Lu Y., Hurwitz E.L., Wu Y. (2022). Gender Disparities of Heart Disease and the Association with Smoking and Drinking Behavior among Middle-Aged and Older Adults, a Cross-Sectional Study of Data from the US Health and Retirement Study and the China Health and Retirement Longitudinal Study. Int. J. Environ. Res. Public Health.

[29] Boakye K., Bovbjerg M., Schuna J., Branscum A., Varma R.P., Ismail R., Barbarash O., Dominguez J., Altuntas Y., Anjana R.M. (2023). Urbanization and physical activity in the global Prospective Urban and Rural Epidemiology study. Sci. Rep..

[30] Kim Y.C., Um Y.J., Yoon S.H., Kim T.W., Seo H.J., Jeong J.H., Hong S.C., Um Y.H. (2024). Association between weekend catch-up sleep and the risk of prediabetes and diabetes: A cross-sectional study using KNHANES. J. Psychosom. Res..

[31] Cinti F., Bouchi R., Kim-Muller J.Y., Ohmura Y., Sandoval P.R., Masini M., Marselli L., Suleiman M., Ratner L.E., Marchetti P. (2016). Evidence of β-Cell Dedifferentiation in Human Type 2 Diabetes. J. Clin. Endocrinol. Metab..

[32] Xu M., Zhou Y., Xu B., Sun J., Wang T., Lu J., Lai S., Bi Y., Wang W., Ning G. (2016). Associations of smoking and alcohol consumption with impaired β-cell function in Chinese men. J. Diabetes.

[33] Kowall B., Rathmann W., Heier M., Holle R., Peters A., Thorand B., Herder C., Strassburger K., Giani G., Meisinger C. (2012). Impact of weight and weight change on normalization of prediabetes and on persistence of normal glucose tolerance in an older population: The KORA S4/F4 study. Int. J. Obes..

[34] Hong S., Chang Y., Jung H.S., Yun K.E., Shin H., Ryu S. (2017). Relative muscle mass and the risk of incident type 2 diabetes: A cohort study. PLoS ONE.

[35] DeFronzo R.A., Tripathy D. (2009). Skeletal muscle insulin resistance is the primary defect in type 2 diabetes. Diabetes Care.

[36] Cai X., Qiu S., Liu S., Lu Y., Luo D., Li R., Li M. (2020). Body-weight fluctuation and risk of diabetes in older adults: The China Health and Retirement Longitudinal Study (CHARLS). Diabetes Res. Clin. Pract..

