# Supplementary material for: Differential Associations of Healthy Lifestyle Latent Classes with Glycemic Transitions in Prediabetes: An Observational Study Based on CHARLS
Source: Healthcare (Basel). 2026 Sep 11;14(18):2966. doi: 10.3390/healthcare14182966 (PMC13606765; doi:10.3390/healthcare14182966)
Supplement: Supplementary file 1 [file healthcare-14-02966-s001.zip › healthcare-4454348-supplementary.pdf]

Supplementary appendix

Contents

Supplementary tables 2

Supplementary figures 14

Supplementary tables

Table S1 Baseline characteristics of the study participants according to glycemic condition

Characteristics	Total sample (n =2529)	Prediabetes to normoglycemia (n=563)	Prediabetes to prediabetes (n=1644)	Prediabetes to diabetes (n=322)	P
**Sociodemographic variables**					
**Age (years)**	59(53,65)	57(49,63) [†‡]	59(53,65) [†§]	60(54,67) [‡§]	**＜0.001**
**Gender**					**0.004**
Male	1136(45.2)	286(51.1) [†‡]	719(43.9) [†]	131(40.9) [‡]	
Female	1380(54.8)	274(48.9)	917(56.1)	189(59.1)	
**Ethnic group**					**0.010**
Han	2364(93.5)	542(96.3) [†]	1524(92.7) [†]	298(92.5)	
Others	165(6.5)	21(3.7)	120(7.3)	24(7.5)	
**Residence**					0.322
Rural	2099(83.0)	470(83.5)	1353(82.3)	276(85.7)	
Urban	429(17.0)	93(16.5)	290(17.7)	46(14.3)	
**Marriage**					0.692
Married and Living with spouse	2152(85.1)	484(86.0)	1398(85.0)	270(83.9)	
Others	382(15.1)	79(14.0)	246(15.0)	52(16.1)	
**Education**					**0.002**
Primary school below	1206(47.7)	234(41.6) [†‡]	797(48.5) [†]	175(54.3) [‡]	
Primary school	573(22.7)	134(23.8)	368(22.4)	71(22.0)	
Middle school or above	749(29.6)	195(34.6)	478(29.1)	76(23.6)	
**Work**					**0.001**
Working	1734(68.6)	412(73.3) [†]	1124(68.5) [‡]	198(61.5) [†‡]	
Not working	792(31.4)	150(26.7)	522(31.5)	124(38.5)	

Table S1 (Continued)

Characteristics	Total sample (n =2529)	Prediabetes to normoglycemia (n=563)	Prediabetes to Prediabetes (n=1644)	Prediabetes to Diabetes (n=322)	P
**Chronic diseases**					
**Hypertension**					**<0.001**
Yes	692(27.5)	144(25.8) †	416(25.5) ‡	132(41.0) †‡	
No	1822(72.5)	415(74.2)	1217(74.5)	190(59.0)	
**Dyslipidemia**					**0.016**
Yes	268(10.8)	57(10.4)	162(10.1) †	49(15.6) †	
No	2203(86.9)	492(89.6)	1445(89.9)	266(84.4)	
**Cognition**	14(9,18)	15(10,19) †	14(9,18)	14(8,18) †	**0.013**
**TC (mg/dL)**	194.27(170.49,219.49)	184.60(163.92,209.54) †‡	197.55(172.71,222.68) †	196.39(173.49,222.58)‡	**<0.001**
**Triglycerides (mg/dL)**	107.97(77.88,158.42)	103.55(73,46,149.34) †	108.86(77.88,178.77) ‡	115.05(82.08,149.34) †‡	**0.006**
**HDL-c(mg/dL)**	49.10(40.21,60.70)	49.48(40.59,60.70)	49.48(40.50,61.08) †	47.35(38.56,58.76) †	**0.033**
**LDL-c(mg/dL)**	117.91(96.26,141.01)	109.99(88.92,131.06) †‡	120.23(88.92.,143,82) †	120.23(100.13,143.04) ‡	**<0.001**
**TyG**	8.67(8.33,9.06)	8.60(8.27,8.98)†	8.67(8.34,9.05) ‡	8.74(8.41,9.19) † ‡	**<0.001**

Note: WC, waist circumference; TC, total cholesterol; LDL-c, low-density lipoprotein-cholesterol; HDL-c, high-density lipoprotein-cholesterol; TyG, Triglyceride-glucose index.

†、‡、§ denote statistically significant differences between pairwise comparisons.

Table S2 Fit statistics models from one to five classes

Model	AIC	BIC	aBIC	LMR LR P-value	ALMR LR P-value	BLRT P-value	Entropy
1C	16367.349	16402.363	16383.299	-	-	-	-
2C	15975.798	16051.661	16010.356	< 0.001	< 0.001	< 0.001	0.549
3C	15933.831	16050.543	15986.998	< 0.001	< 0.001	< 0.001	0.676
4C	15914.604	16072.165	15986.378	0.049	0.052	< 0.001	0.509
5C	15912.687	16111.097	16003.070	0.054	0.057	0.091	0.626

Note: AIC-Akaike Information Criterion; BIC-Bayesian Information Criterion; aBIC-Adjusted BIC; LMR LR- Lo- Mendell-Rubin likelihood ratio; ALMR LR- Adjusted LMR LR; BLRT- bootstrap likelihood ratio test

-: Not applicable.

Table S3 Average latent class probabilities for most likely latent class membership by latent class (column)

Most Likely Class (Row)	Class 1	Class 2	Class 3
Class 1	**0.804**	0.160	0.036
Class 2	0.116	**0.816**	0.067
Class 3	0.094	0.002	**0.905**

Note: Values in bold on the diagonal represent the average posterior probability of individuals assigned to each class actually belonging to that class. Higher diagonal values indicate better classification accuracy. The off-diagonal values represent the probability of being misclassified into another class.

Table S4 The latent-class–conditional probability distributions for the four-class model.

Healthy lifestyle		Conditional Probability by Class			
		CLASS1 (n=570，22.5%)	CLASS2 (n=433，17.1%)	CLASS 3 (n=537，21.2%)	CLASS4 (n=989, 39.1%)
BP	hypertension	0.000	0.356	0.532	0.326
	Not hypertension	1.000	0.644	0.468	0.674
Sleep duration	<6h;>9h	0.323	0.131	0.458	0.349
	6-9h	0.677	0.869	0.542	0.651
Smoking habits	Smoking	0.116	0.066	0.076	0.618
	Not smoking	0.884	0.934	0.924	0.382
Alcohol consumption	Drinking	0.103	0.252	0.000	0.730
	Not drinking	0.897	0.748	1.000	0.270
Physcial activity	Not Meet guidelines	0.218	0.356	0.563	0.272
	Meet guidelines	0.782	0.644	0.437	0.728
BMI	≥25	0.092	1.000	0.441	0.205
	＜25	0.908	0.000	0.559	0.795

Note: BP, systolic blood pressure; BMI, bodymass index (calculated as weight in kilograms divided by square of height in meters).

Table S5 The latent-class–conditional probability distributions for the three-class model

Healthy lifestyle		Conditional Probability by Class		
		CLASS1 (n=697，27.6%)	CLASS2 (n=712，28.2%)	CLASS 3 (n=1120，44.3%)
BP	hypertension	0.241	0.402	0.306
	Not hypertension	0.759	0.598	0.694
Sleep duration	<6h;>9h	0.383	0.288	0.327
	6-9h	0.617	0.712	0.673
Smoking habits	Smoking	0.135	0.081	0.672
	Not smoking	0.865	0.919	0.328
Alcohol consumption	Drinking	0.111	0.149	0.789
	Not drinking	0.889	0.851	0.211
Physcial activity	Not Meet guidelines	0.382	0.416	0.243
	Meet guidelines	0.618	0.584	0.757
BMI	≥25	0.000	1.000	0.214
	＜25	1.000	0.000	0.786

Note: BP, systolic blood pressure; BMI, bodymass index (calculated as weight in kilograms divided by square of height in meters).

Table S6 Association between healthy lifestyle factors and transition of glycemic status

Healthy lifestyle		Prediabetes to Normoglycemia		Prediabetes to Diabetes	
		OR	P	OR	P
HBP	hypertension	0.770 (0.527,1.126)	0.849	1.262(0.814,)	0.327
	Not hypertension	1(Ref.)		1(Ref.)	
Sleep duration	<6h;>9h	1.025 (0.713,1.473)	0.909	1.120 (0.717,1,749)	0.637
	6-9h	1(Ref.)		1(Ref.)	
Smoking habits	Smoking	0.987 (0.630,1.547)	0.959	1.039 (0.571,1.890)	1.000
	Not smoking	1(Ref.)		1(Ref.)	
Alcohol consumption	Drinking	0.745 (0.490,1.133)	0.137	0.921 (0.530,1.601)	1.000
	Not drinking	1(Ref.)		1(Ref.)	
Physical activity	Not Meet guidelines	0.937 (0.646,1.360)	0.702	0.779 (0.487,1.247)	0.212
	Meet guidelines	1(Ref.)		1(Ref.)	
BMI	≥25	0.635(0.431,0.934)	**0.021**	1.927(1.210,3.067)	**0.005**
	＜25	1(Ref.)		1(Ref.)	

a Adjusted for age, gender, place of residence, marital status, educational level, working, presence of dyslipidemia, presence of CVD and cognition.

Multiple imputation was used for missing covariates, under the assumption that data were Missing at Random.

OR, Odds Ratio; HBP, high blood pressure; BMI, body mass index.

Table S7 Sensitivity analysis of the association between healthy lifestyle classes and glycemic change.

	Unadjusted model				adjusted model [a]			
	Prediabetes to Normoglycemia		Prediabetes to Diabetes		Prediabetes to Normoglycemia		Prediabetes to Diabetes	
	OR	P	OR	P	OR	P	OR	P
Class 1[b]	0.853(0.680, 1.070)	0.169	0.973(0.706, 1.341)	**0.866**	0.736(0.554, 0.978)	**0.034**	1.110(0.746, 1.650)	0.607
Class 2[b]	0.694(0.544, 0.884)	**0.003**	2.051(1.588, 2.701)	**<0.001**	0.712(0.531, 0.955)	**0.024**	2.469(1.797, 3.393)	**<0.001**
Class 3[b]	1(Ref.)		1(Ref.)		1(Ref.)		1(Ref.)	

Note:

a Adjusted for age, gender, place of residence, marital status, educational level, presence of dyslipidemia and working.

b Class 1=smoking and drinking group health risk group; Class 2= high-BMI group; Class 3=healthy lifestyle group.

Using the original data without imputation.

OR, Odds Ratio.

Table S8 Baseline characteristics of 182 individuals with prediabetes.

Characteristics		n/x±s/M[P25,P75]	Proportion （%）
Sociodemographic variables			
Gender	Male	66	36.3
	Female	116	63.7
Age(year)	18~44	100	55.2
	45~59	51	28.2
	≥60	30	16.6
Education	Junior high school or below	21	11.6
	High school and junior college	58	32.0
	Bachelor's degree or above	102	56.4
Per-capita monthly household income	≤6000	32	20.8
	＞6000	122	79.2
Marriage	Married and Living with spouse	153	84.1
	Others<	28	15.4
Residence	Urban	177	97.3
	Rural	5	2.7
Work	heavy-physical-labor	7	3.8
	middle-physical-labor	17	9.3
	light-physical-labor	122	67.0
	heavy-physical-labor	36	19.8
medical payment	medical insurance	177	97.3
	Commercial insurance or self-payment	5	2.7
Family history of diabetes	Yes	58	31.8
	no	124	68.1
Number of other chronic diseases	0	53	29.1
	1	63	34.6
	≥2	66	23.7
hypoglycemic drugs	Yes	53	29.1
	No	129	70.9
smoking	Yes	22	12.1
	No	160	87.9
drinking	Yes	23	12.6
	no	159	87.4
Sleep duration	6~9	148	81.3
	＜6 or ＞9	34	18.7
Self-rated health status	fairly good	43	23.6

	Ordinary	92	50.5
	Poor	47	25.8
BMI(kg/m^2)		27.33±5.25	
FBG(mmol/L)		5.70(5.10,5.50)	
HbA1c(%)		5.80(5.50,6.10)	
2h OGTT(mmol/L)		8.94±2.19	

Note: The 182 patients were enrolled at a tertiary hospital in Shanghai for external validation, and the study has obtained ethical approval from Shanghai University of Traditional Chinese Medicine.

The difference from CHARLS-based studies is that the participants are younger, the vast majority live in urban areas, and there is a higher level of education.

Table S9 Fit statistics models from one to five classes of 182 individuals with prediabetes.

Model	AIC	BIC	aBIC	LMR LR P-value	ALMR LR P-value	BLRT P-value	Entropy
1C	1070.149	1089.373	1070.370	-	-	-	-
2C	1066.974	1108.626	1067.453	0.012	0.013	0.040	1.000
3C	1071.354	1135.434	1072.092	0.3087	0.3202	0.6667	0.950
4C	1077.600	1164.108	1078.595	0.168	0.176	0.667	0.797
5C	1088.447	1197.701	1089.701	0.349	0.355	1.000	0.808

Note: The 182 patients were enrolled at a tertiary hospital in Shanghai for external validation, and the study has obtained ethical approval from Shanghai University of Traditional Chinese Medicine.

AIC-Akaike Information Criterion; BIC-Bayesian Information Criterion; aBIC-Adjusted BIC; LMR LR- Lo- Mendell-Rubin likelihood ratio; ALMR LR- Adjusted LMR LR; BLRT- bootstrap likelihood ratio test

-: Not applicable.

Table S10 The latent-class–conditional probability distributions of 182 individuals with prediabetes.

Healthy risk factors		Conditional Probability by Class		
		CLASS1 (n=127，69.8%)	CLASS2 (n=18，10.4%)	CLASS 3 (n=36，19.8%)
BP	hypertension	0.234	0.264	0.193
	Not hypertension	0.776	0.736	0.807
Sleep duration	<6h;>9h	0.252	**0.000**	0.027
	6-9h	0.748	1.000	0.973
Smoking habits	Smoking	0.052	**1.000**	0.021
	Not smoking	0.948	0.000	0.979
Alcohol consumption	Drinking	0.112	0.438	0.055
	Not drinking	0.888	0.562	0.945
Physical activity	Not Meet guidelines	**1.000**	0.638	**0.000**
	Meet guidelines	0.000	0.362	1.000
BMI	≥25	**0.676**	0.710	0.517
	＜25	0.324	0.290	0.483

Note: The 182 patients were enrolled at a tertiary hospital in Shanghai for external validation, and the study has obtained ethical approval from Shanghai University of Traditional Chinese Medicine.

BP, systolic blood pressure; BMI, bodymass index (calculated as weight in kilograms divided by square of height in meters).

Supplementary figures

Figure S1 Blood glucose transition patterns in CHARLS in the 4-year period

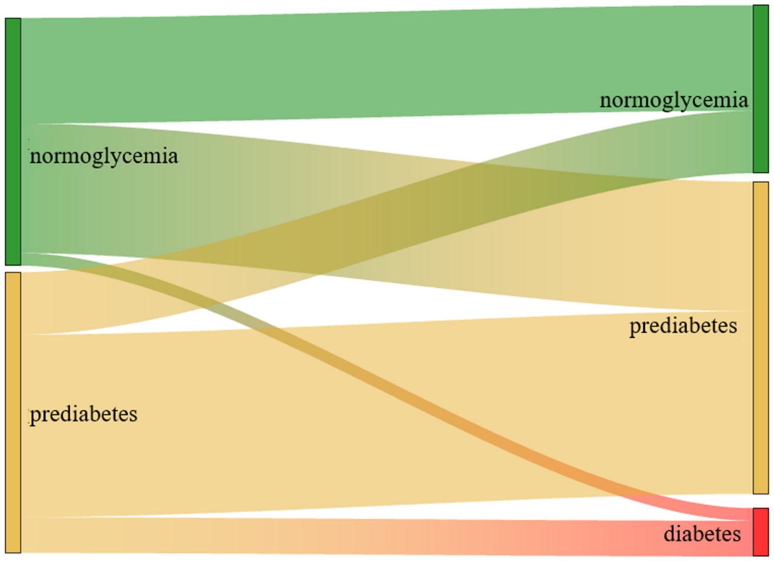

† The Sankey diagram uses data from two waves (2011 and 2015), illustrating the blood glucose changes in the non-diabetic population over the four-year period.

‡ Among participants with prediabetes at baseline: 1,644 (65%) remained prediabetic at wave 3;

322 (12%) progressed to diabetes; and 563 (22%) reverted to normoglycemia. Among participants with normoglycemia at baseline: 1,172 (52%) developed prediabetes at wave 3;

114 (5%) progressed directly to diabetes; while 954 (43%) maintained normoglycemia at wave 3.

Figure S2 Data Missingness Pattern

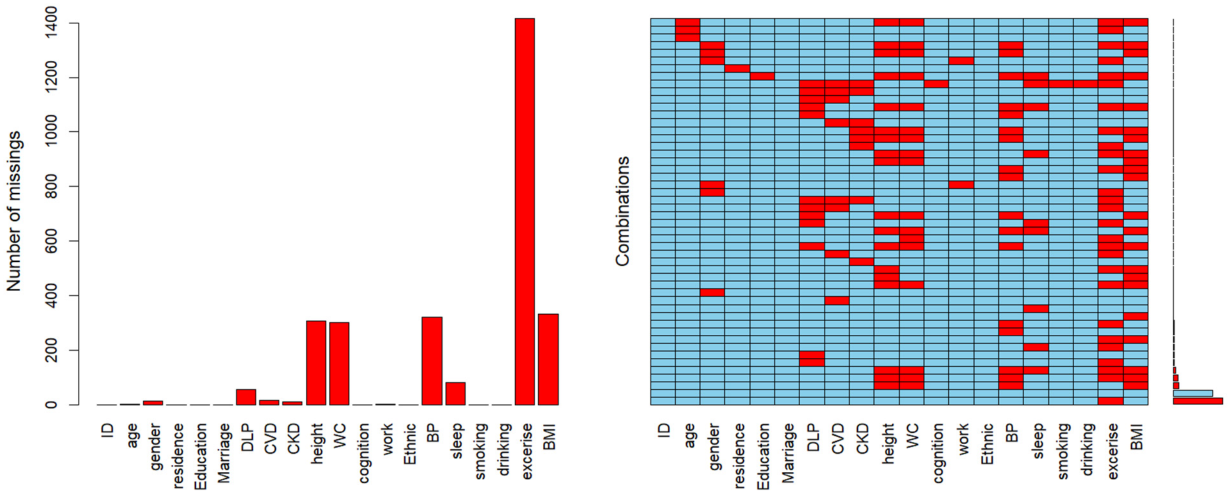

† BP, Blood Pressure; DBP, Diastolic Blood Pressure ;BMI, body mass index (calculated as weight in kilograms divided by square of height in meters)； CDK, Chronic Kidney Disease; CVD, Cardiovascular Disease; DLP, Dyslipidemia; WC, Waist Circumference.
